# Validierung und Reliabilitätsprüfung des Nijmegen Cochlear Implant Questionnaire in deutscher Sprache

**DOI:** 10.1007/s00106-021-01114-0

**Published:** 2021-10-14

**Authors:** Michaela Plath, Matthias Sand, Philipp S. van de Weyer, Kilian Baierl, Mark Praetorius, Peter K. Plinkert, Ingo Baumann, Karim Zaoui

**Affiliations:** 1 Universitäts-HNO-Klinik Heidelberg, Im Neuenheimer Feld 400, 69120 Heidelberg, Deutschland; 2 GESIS-Leibniz-Institut für Sozialwissenschaften, Mannheim, Deutschland; 3 Universitäts-HNO-Klinik Hamburg, Martinistrasse 52, Gebäude O10, 20246 Hamburg, Deutschland

**Keywords:** Lebensqualität, Cochleaimplantation, NCIQ, Validität, Reliabilität, Quality of life, Cochlear implantation, NCIQ, Validity, Reliability

## Abstract

**Hintergrund:**

Der Nijmegen Cochlear Implant Questionnaire (NCIQ) ist ein krankheitsspezifischer Fragebogen zur Erhebung der gesundheitsbezogenen Lebensqualität von Patienten vor und nach Cochleaimplantation.

**Ziel der Arbeit:**

Validierung und Reliabilitätsprüfung der deutschen Übersetzung des NCIQ.

**Material und Methoden:**

Es wurde eine prospektive Studie an 100 postlingual ertaubten oder hochgradig schwerhörigen Patienten durchgeführt, welche präoperativ sowie 3 und 6 Monate nach einer Cochleaimplantation mittels NCIQ, Abbreviated Profile of Hearing Aid Benefit (APHAB) und Hearing Participation Scale (HPS) untersucht wurden. Als Kontrolle fungierte ein postlingual ertaubtes oder hochgradig schwerhöriges, unbehandeltes Patientenkollektiv (*n* = 54). Cronbach‑α und Test-Retest-Reliabilität dienten der Reliabilitätsüberprüfung. Es wurde auf Inhalts‑, Übereinstimmungs- und auf diskriminative Validität getestet. Die Konstruktvaliditätsprüfung basiert auf kürzlich veröffentlichen Daten. Als Gütekriterien wurden die Sensitivität und eine ROC(„Receiver Operating Characteristic“)-Analyse, inklusive AUC(„Area Under the ROC Curve“)-Betrachtung, eingesetzt.

**Ergebnisse:**

Das Test-Retesting ergab nach 3 und 6 Monaten postoperativ stabile NCIQ-Werte. Die Cronbach-α-Werte wiesen auf eine gute interne Konsistenz hin. Der NCIQ diskriminierte valide zwischen behandelten und unbehandelten Patientengruppen. Es ergaben sich statistisch signifikante, wenn auch schwache, Korrelationen zwischen dem NCIQ und dem APHAB (r = −0,22; *p* = 0,04) und dem HPS (r = 0,30; *p* = 0,01). Sensitivitäts- und ROC-Analysen zeigten eine gute Messqualität des deutschsprachigen NCIQ.

**Schlussfolgerung:**

Die deutsche Übersetzung des NCIQ misst zuverlässig und valide die Lebensqualität vor und nach Cochleaimplantation und kann zur klinischen Erfolgskontrolle nach Cochleaimplantationen verwendet werden.

## Abstract (en)

**Background:**

The Nijmegen Cochlear Implant Questionnaire (NCIQ) is a disease-specific questionnaire to determine the health-related quality of life (HRQoL) of patients before and after cochlear implantation.

**Objective:**

This study aimed to assess the validity and reliability of the German translation of the NCIQ.

**Materials and methods:**

A prospective study was performed in 100 postlingually deaf or severely hearing-impaired patients. HRQoL was assessed using the NCIQ, the Abbreviated Profile of Hearing Aid Benefit (APHAB), and the Hearing Participation Scale (HPS) before as well as 3 and 6 months after cochlear implantation. An untreated group of postlingually deaf or severely hearing-impaired patients (*n* = 54) served as a control. Cronbach’s α and test–retest reliability were measured. The content, discrimination, and agreement validity were tested. The evaluation of construct validity was based on recently published data. Sensitivity and receiver operating curve (ROC) analysis, including consideration of the area under the curve (AUC), were used as quality criteria.

**Results:**

The test–retest analysis showed stable NCIQ values 3 and 6 months postoperatively. The Cronbach’s α values indicated good internal consistency. The NCIQ validly discriminated between treated and untreated patient groups. There were statistically significant albeit weak correlations between the NCIQ and the APHAB (r = −0.22; *p* = 0.04) and the HPS (r = 0.30; *p* = 0.01). Sensitivity and ROC analyses showed good measurement quality of the German-speaking NCIQ.

**Conclusion:**

The German translation of the NCIQ reliably and validly measures HRQoL before and after cochlear implantation and can be used for clinical monitoring after treatment with cochlear implants.

Zur Messung der gesundheitsbezogenen Lebensqualität nach Cochleaimplantation finden neben der audiologischen Erfolgskontrolle verschiedene validierte Messinstrumente Anwendung. In der neuen S2k-Leitlinie (AWMF-Register-Nr. 017/071AWMF-Register-Nr. 017/071; Titel: Cochlea-Implantat Versorgung; https://www.awmf.org/uploads/tx_szleitlinien/017-071l_S2k_Cochlea-Implantat-Versorgung-zentral-auditorische-Implantate_2020-12.pdf) wird die Verwendung des Nijmegen Cochlear Implant Questionnaire (NCIQ) im Rahmen einer Cochleaimplantatversorgung empfohlen. Das macht eine Validierung und Reliabilitätsprüfung der deutschen Übersetzung des NCIQ unabdingbar.

## Hintergrund und Fragestellung

Jährlich werden in Deutschland etwa 4000 Patienten mit Cochleaimplantaten versorgt [57]. Die Zahl der Cochleaimplantationen hat in den letzten Jahren stark zugenommen [7, 14]. Aufgrund der technischen Weiterentwicklung ist bei den Betroffenen zunehmend ein offenes Sprachverständnis erzielt worden [25]. Hierdurch wurde es vielen hochgradig Schwerhörigen und an Taubheit grenzenden Schwerhörigen wieder ermöglicht, am sozialen und beruflichen Leben teilzunehmen [24, 35].

In einigen Studien wurde gezeigt, dass es infolge einer Cochleaimplantation zu Verbesserungen der Hörfähigkeit, der Sprachwahrnehmung und der Sprachproduktion kommt [26, 29, 34, 55]. Allerdings spiegeln die künstlichen Testbedingungen oft nicht die tatsächlichen Hörsituationen wider, welchen die Cochleaimplantat(CI)-Träger in ihrem sozialen Umfeld ausgesetzt sind. So stellen das Hören im Störschall oder widerhallender Umgebung für Menschen mit CI weiterhin erschwerte Umweltbedingungen dar.

In den letzten Jahren hat der Einsatz von „Patient-Reported Outcomes“ (PRO) zur Erfassung der gesundheitsbezogenen Lebensqualität („Health-Related Quality of Life“, HRQoL) nach Cochleaimplantation zunehmend an Bedeutung gewonnen. In der Literatur konnte dabei eine signifikante Verbesserung der Lebensqualität nach Cochleaimplantation nachgewiesen werden [13, 27, 33]. Hierbei zeigte sich der Nijmegen Cochlear Implant Questionnaire (NCIQ) als sensitives und zuverlässiges Lebensqualitätsmessinstrument nach Cochleaimplantation, welches von Hinderink et al. im Jahr 2000 entwickelt und in englischer Sprache validiert wurde. Der NCIQ testet nicht nur die physischen und funktionellen Dimensionen, sondern deckt zusätzlich auch die psychosozialen Veränderungen nach Cochleaimplantation auf [25]. Der NCIQ wurde in viele verschiedene Sprachen übersetzt. Nach Validierung der chinesischen Version (Nijmegen人工耳蜗植入量表中文版信度和效度评价) durch die Arbeitsgruppe Dong et al. im Jahr 2010 [15] folgten die Validierungen der spanischen Version (Fiabilidad y validez del Cuestionario de implante coclear de Nijmegen en español) durch Sanchez-Cuadrado et al. im Jahr 2014 [50] und der italienischen Version (Adattamento interculturale e convalida del questionario sull’impianto cocleare di Nijmegen in italiano) durch Ottaviani et al. im Jahr 2016 [43].

Zur Sicherung der Ergebnisqualität nach Cochleaimplantation werden neben den psychoakustischen Verfahren zum Hörstatus und Sprachverstehen validierte Messinstrumente zur Bewertung des subjektiv unterschiedlich empfundenen Gesundheitszustands und der Lebensqualität benötigt. Validierte Lebensqualitätsmessinstrumente sind fester Bestandteil in der jüngst überarbeiteten S2k-Leitlinie zur Durchführung einer Cochleaimplantatversorgung (S2k-Leitlinie; AWMF-Register-Nr. 017/071; Titel: Cochlea-Implantat Versorgung; https://www.awmf.org/uploads/tx_szleitlinien/017-071l_S2k_Cochlea-Implantat-Versorgung-zentral-auditorische-Implantate_2020-12.pdf).

Folglich ist die Validierung und Reliabilitätsprüfung der deutschen Übersetzung des NCIQ an einem Patientenkollektiv mit Deutschkenntnissen auf muttersprachlichem Niveau unabdingbar und stellt das Ziel dieser Arbeit dar.

## Studiendesign und Untersuchungsmethoden

### „Patient-Reported Outcomes“ (PRO)

#### Nijmegen Cochlear Implant Questionnaire (NCIQ)

Im Jahr 2000 publizierten und validierten Hinderink et al. den NCIQ in englischer Sprache [25]. Der aus 60 Einzelfragen bestehende Fragebogen testet drei verschiedene Hauptdomänen mit insgesamt sechs Subdomänen:

1. *physische Hauptdomäne (Physical Functioning)*
  mit den drei Subdomänen:
  a) elementare Geräuschwahrnehmung (Sound Perception Basic)
  b) fortgeschrittene Geräuschwahrnehmung (Sound Perception Advanced)
  c) Sprachproduktion (Speech Production)
2. *psychische Hauptdomäne (Psychological Functioning)*
  mit der Erhebung des Selbstwertgefühls (Self-Esteem)
3. *soziale Hauptdomäne (Social Functioning)*
  mit den zwei Subdomänen:
  a) Aktivitätseinschränkung (Activity)
  b) soziale Interaktion (Social Interaction)

**Figure 1:**
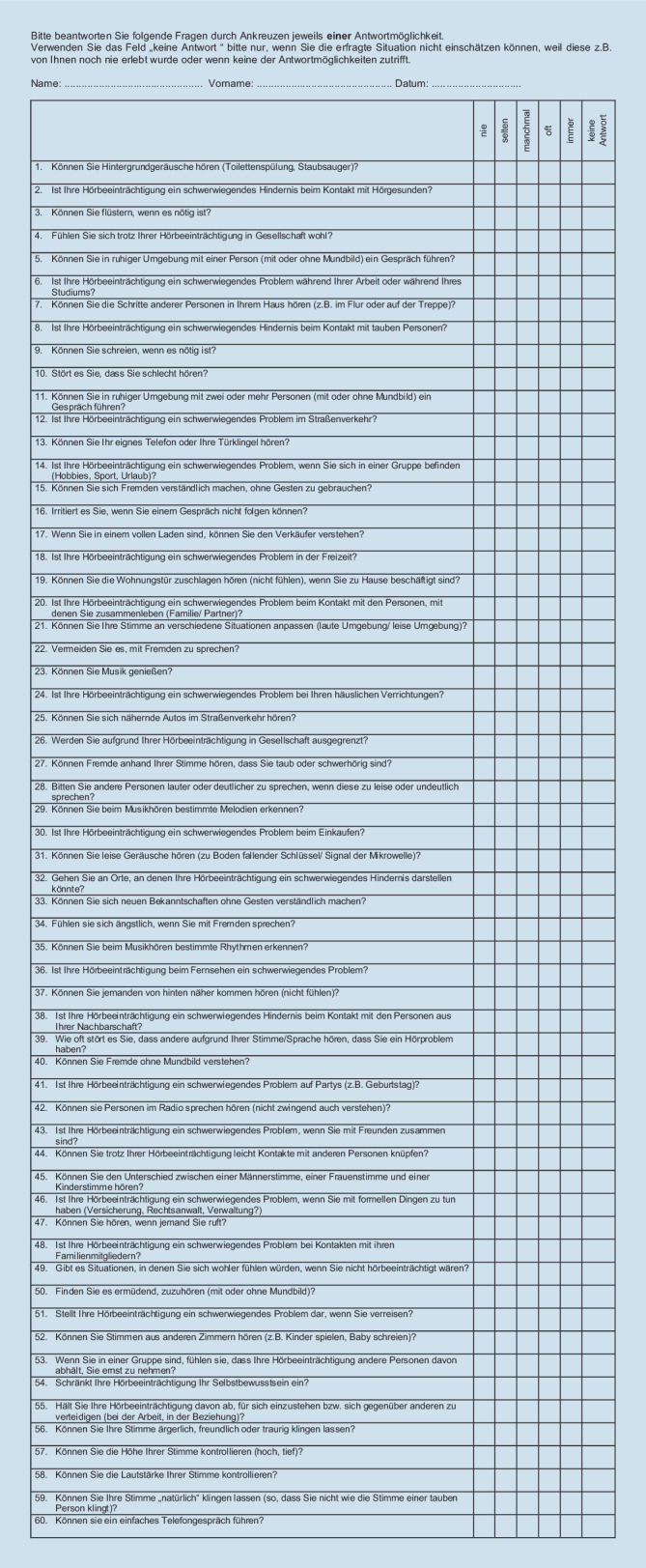

Die deutsche Übersetzung des NCIQ (Abb. 1) wurde uns freundlicherweise von der Arbeitsgruppe von Frau Prof. H. Olze (Charité – Universitätsmedizin Berlin) zur Verfügung gestellt und ist bereits in Studien eingesetzt worden [27, 32, 41]. Die Patienten unserer Studie hatten 60 Einzelfragen mit sechs Antwortmöglichkeiten „nie“, „selten“, „manchmal“, „oft“ und „immer“ oder „keine Antwort“ zu beantworten. Die Markierung der Antwortmöglichkeit „keine Antwort“ führte, wie auch die Nichtbeantwortung, zum Ausschluss der Frage. Wie in der Originalveröffentlichung von Hinderink et al. [25] beschrieben, wurden die fünf Antwortkategorien für alle Elemente wie folgt transformiert: „nie“ entspricht einem Punktewert von 0, „selten“ einem Wert von 25, „manchmal“ einem Wert von 50, „oft“ einem Wert von 75 und „immer“ einem Wert von 100. Siebenundzwanzig Fragen sind im Fragebogen invers formuliert, sodass die Antworten bei der Auswertung umcodiert werden mussten. Die Fragen sind in 6 Subdomänen mit je 10 Fragen unterteilt. Die Subdomänenwerte ergeben sich aus der Addition der Antworten der 10 Fragen, geteilt durch die Anzahl der ausgefüllten Fragen. Die Antworten wurden auf einer 5‑Punkte-Likert-Skala mit Punktzahlen zwischen 0 (sehr schlecht) und 100 (optimal) berechnet. Eine höhere Punktezahl spiegelt eine bessere subjektive Bewertung der Hörleistung und eine niedrigere Punktzahl entsprechend ein ausgeprägtes Hörhandicap wider [49]. Die jeweilige Zuteilung der Fragen zu den Haupt- und Subdomänen sind in der im Jahr 2000 publizierten Originalveröffentlichung von Hinderink et al. als Codebuchanhang zu finden. Im Jahr 2017 wurde der Codebuchanhang allerdings korrigiert und als Corrigendum veröffentlicht. Unsere Datenanalyse des NCIQ erfolgte gemäß des 2017 erschienenen Corrigendums des Codebuchanhangs [25]. Hierbei wurden die initial zur Subdomäne „Advanced Sound Perception“ zugeordneten Fragen mit den Fragen der Subdomäne „Speech Production“ getauscht. Zusätzlich sollte das Item 53 der Subdomäne „soziale Interaktionen“ auch in der Spalte „Recoding“ aufgeführt werden.

Im Rahmen der Validierung der deutschsprachigen Version des NCIQ beantworteten alle Studienteilnehmer zwei weitere „patient-reported outcomes“ (PRO) zur Messung der gesundheitsbezogenen Lebensqualität.

#### Abbreviated Profile of Hearing Aid Benefit (APHAB)

Der APHAB besteht aus 24 Fragen, die sich in vier Subkategorien mit je sechs Fragen unterteilen: Leichtigkeit der Kommunikation („ease of communication“, EC), Kommunikation in halliger, echoreicher Umgebung („reverberation“, RV), Kommunikation bei Hintergrundgeräuschen („background noise“, BN) und Abneigung („aversiveness“, AV). Die Ergebnisse des APHAB werden in drei Stufen eingeteilt: 0–16 Punkte: „kein Handicap“, 18–42 Punkte: „leichtes bis mäßiges Handicap“ und mehr als 42 Punkte: „schweres Handicap“ [58]. Die Validierung des deutschsprachigen Fragebogens APHAB liegt nur für dessen Verwendung mit Hörgeräten vor [49], nicht aber für die Verwendung im Rahmen der CI-Versorgung, und findet in klinischen Studien im Einsatz mit Hörgeräten häufig Verwendung [36].

#### Hearing Participation Scale (HPS)

Der von Hawthorne et al. entwickelte HPS ist die gekürzte Version des Glasgow Health Status Inventory [23] und besteht aus elf Fragen, welche die drei Hördimensionen: Selbstwertgefühl bezüglich des Hörens („self-esteem to hearing“), Sozialintegrationsniveau („level of social integration“) und Hörbeeinträchtigung („hearing handicap“) umfassen. Obgleich derzeit noch eine Validierung der deutschen Version des HPS aussteht [4], findet der HPS aufgrund seiner Vorzüge als postoperativ einzusetzender [37], CI-spezifischer Fragebögen [49], der alle drei Hauptdomänen des NCIQ abdeckt, hier Verwendung. Wie auch beim NCIQ signalisieren höhere Fragebogenwerte ein besseres Outcome [23].

### Ethische Begutachtung

Die Ethikkommission der Medizinischen Fakultät der Universität Heidelberg erteilte die Erlaubnis zur Durchführung der Studie (Projekt Nr. S‑481/2009). Die Studie wurde gemäß der Deklaration von Helsinki zur biomedizinischen Forschung an Menschen durchgeführt. Alle Patienten wurden über die Studienziele und das Studienprotokoll informiert und die Studienteilnehmer wurden nach schriftlicher Einwilligung in die Studie aufgenommen. Die Studienteilnahme war freiwillig und unentgeltlich.

### Rekrutierung der Studiengruppen

Die hier rekrutierten Studienteilnehmer lassen sich entsprechend ihrer Datensätze in unterschiedliche Gruppen einteilen: CI-Gruppe präoperativ (*n* = 100), CI-3M-Gruppe (*n* = 85), CI-6M-Gruppe (*n* = 65) und die Kontrollgruppe (*n* = 54).

#### CI-Gruppe präoperativ

In diese Studie wurden initial 100 postlingual ertaubte, an Taubheit grenzende schwerhörige oder hochgradig schwerhörige Patienten mit Deutschkenntnissen auf muttersprachlichem Niveau eingeschlossen, bei denen zwischen Januar 2011 und Dezember 2013 eine Cochleaimplantation an der Universitäts-HNO-Klinik Heidelberg durchgeführt wurde. Die erste Datenerhebung erfolgte präoperativ. Einschlusskriterien der Studienteilnahme waren eine ein- oder beidseitige Ertaubung, eine hochgradig bzw. an Taubheit grenzende Schwerhörigkeit und die Volljährigkeit (> 18 Jahre). Die hier verwendeten Implantatsysteme waren von MED-EL, Cochlear oder Advanced Bionics und wurden alle von einem der Autoren (Ma. P.) implantiert.

#### CI-Gruppen postoperativ

Neben der präoperativen Datenerhebung erfolgten bei der CI-Gruppe präoperativ auch postoperative Messungen zu zwei weiteren definierten Messzeitpunkten: nach drei (CI-3M-Gruppe; *n* = 85) und nach sechs Monaten (CI-6M-Gruppe; *n* = 65) postoperativ.

Bedingt durch fehlende Rückantworten (Verweigerung der Wiederbefragung, Umzug, Incompliance) verminderte sich die Studienteilnehmeranzahl während des postoperativen Untersuchungszeitraums.

#### Kontrollgruppe

Als Kontrollgruppe wurde an der Universitäts-HNO-Klinik im Zeitraum von Ende 2016 bis Ende 2019 ein gleichermaßen erkranktes und noch unbehandeltes Patientenkollektiv (*n* = 54) mit Deutschkenntnissen auf muttersprachlichem Niveau rekrutiert. In Anlehnung an die Originalveröffentlichung von Hinderink et al. [25] handelte es sich bei der Kontrollgruppe um 54 postlingual gehörlose, an Taubheit grenzende oder hochgradig schwerhörige Patienten, die bei entsprechender Indikation zur Cochleaimplantation in unsere Studie integriert wurden. Voraussetzung für den Einschluss in die Kontrollgruppe war eine Ertaubung sowie eine ähnliche Alters- und Geschlechtsverteilung wie die CI-Gruppe präoperativ. Die Patienten der Kontroll- und der CI-Gruppe präoperativ nahmen an einer einmal wöchentlich stattfindenden Cochleaimplantat-Sprechstunde teil und wurden nach Erfüllen der Hör- und bildgebenden diagnostischen Kriterien sowie nach Beurteilung der Hörrehabilitationsfähigkeit zu einer Cochleaimplantation zugelassen. Die Studienteilnehmer-Rekrutierung fand im Rahmen des präoperativen Aufklärungsgespräches statt, als die Entscheidung zur Cochleaimplantation bereits getroffen war. Die Evaluation der jeweiligen Hörleistungen war nicht Gegenstand unserer Datenanalyse.

### Validität- und Reliabilitätsprüfung der englischen Version des NCIQ

In der Originalveröffentlichung von Hinderink et al. auf englischer Sprache wurden 45 postlingual ertaubte Patienten, welche im Zeitraum von 1989 bis 1997 cochleaimplantiert wurden, in die Studie integriert. Als Kontrollgruppe (Baseline) fungierten 46 postlingual gehörlose Patienten, die sich auf der Warteliste des Institutes für eine Cochleaimplantation befanden. Die prä- und postoperativen Subdomänen-NCIQ-Werte wurden jeweils mit denen der Kontrollgruppe verglichen, wobei die präoperativen NCIQ-Werte similär zu denen der Kontrollgruppe ausfielen. Die psychometrische Testung des NCIQ erfolgte zum einen durch die Bewertung des Grades an Übereinstimmung (interne Konsistenz) der sechs NCIQ-Domänen unter Verwendung von Cronbach‑α, wobei ein α‑Koeffizient von 0,70 oder höher als akzeptabel angesehen wurde. Zum anderen wurde die englische Version des NCIQ den Studienteilnehmern zu zwei verschiedenen Zeitpunkten vorgelegt (präoperativ und 2 Monate postoperativ), um die Retest-Reliabilität zu bestimmen. Um die Kriteriumsvalidität zu bewerten, wurden die Ergebnisse der 6 Subdomänen des englischsprachigen NCIQ mit den Antworten der Hörleistungstests (Spondee and Environmental Sounds Identification Tests) in Beziehung gesetzt, wobei keine Korrelationen zwischen den Ergebnissen dieser Hörwahrnehmungstests und den Ergebnissen des NCIQ gefunden wurden. Zur Konstruktvaliditätsuntersuchung wurde eine konfirmatorische Faktorenanalyse („confirmatory factor analysis“) durchgeführt. Die Übereinstimmungsvalidität wurde mit zwei generischen Lebensqualitätsmessinstrumenten überprüft [25]. Ob die englische Version des NCIQ tatsächlich Änderungen registriert, wurde mittels einer Reaktionsindex-Schätzung („responsiveness index“) berechnet. Hierbei wurden die Ergebnisveränderungen prä- zu postoperativ durch die Variabilität der stabilen Test-Retest-Ergebnisse dividiert.

### Validität- und Reliabilitätsprüfung der deutschen Version des NCIQ

Fragebögen zur Messung der gesundheitsbezogenen Lebensqualität spielen eine wichtige Rolle in Forschung, klinischer Praxis und Gesundheitsbewertung. Zuverlässigkeit (Reliabilität) und Gültigkeit (Validität) werden als die wichtigsten Testgütekriterien solcher Messinstrumente angesehen. Das Gütekriterium der Reliabilität betrifft die Messgenauigkeit eines Tests und zeigt auf, ob das Testergebnis tatsächlich zeitlich und räumlich konsistent zu reproduzieren ist. Um das Ausmaß der Reliabilität zu bestimmen, wurden im Rahmen der klassischen Testtheorie mehrere Verfahren hierzu entwickelt, solche wie die Retest-Reliabilität, Paralleltest-Reliabilität, Testhalbierungs-Reliabilität und die innere Konsistenz [39]. In unserer Studie findet die Retest-Reliabilitäts- und die innere Konsistenzprüfung Anwendung. Das Gütekriterium der Validität befasst sich mit der inhaltlichen Übereinstimmung zwischen dem vom Test gemessenen Merkmal und dem Merkmal, welches man messen will [38, 52]. Um ein differenziertes Bild der Gültigkeit der deutschen Version des NCIQ zu erhalten, wird in unserer Studie die Inhalts‑, Übereinstimmungs- und differenzielle Validität untersucht. Die Prüfung der Konstruktvalidität basiert auf kürzlich erschienenen Daten von Plath et al. [45].

In Anlehnung an die Originalveröffentlichung von Hinderink et al. [25] fanden in unserer Studie zur Validierung und Reliabilitätsprüfung der deutschen Übersetzung des NCIQ folgende statistische Methoden Anwendung:

#### Reliabilität.

Um die Reliabilität nach dem *Retest-Verfahren* zu bestimmen, wird ein und derselbe Test (hier: deutsche Version des NCIQ) den Studienteilnehmern zu zwei verschiedenen Zeitpunkten vorgelegt und die Reliabilität wird dann als Korrelation zwischen den beiden Testergebnissen ermittelt [38]. In unserer Studie wurden den Studienteilnehmern drei und sechs Monate nach Cochleaimplantation die deutsche Version des NCIQ, der APHAB sowie der HPS ausgehändigt. Anschließend wurden die Ergebnisse mittels parametrischer Korrelationsanalyse nach Pearson miteinander korreliert.

Die interne Konsistenz des Fragebogens wurde durch das *Cronbach‑α* für den Gesamtwert und die drei Haupt- und sechs Subdomänen ermittelt. Cronbach‑α wird verwendet, um den Grad an Übereinstimmung (interne Konsistenz) zwischen mehreren Fragen in einem Fragebogen zu messen [11]. Ist Cronbach-α ≥ 0,7, wird von einer hohen internen Konsistenz des Instruments ausgegangen [11].

#### Validität.

Die Prüfung auf Validität erfolgte in Anlehnung an bereits veröffentlichte Fragebogen-Validitätsstudien unter Einschluss der *Inhaltsvalidität* in Form einer Literaturrecherche [3, 15, 43]. Zudem bezeichnet die Inhaltsvalidität einen Teilaspekt der Konstruktvalidität und liegt vor, wenn die Messungen eines Konstrukts dessen Inhalt vollständig erfassen [54]. Die Inhaltsvalidität wird in der Regel nicht numerisch anhand eines Maßes bzw. Kennwerts bestimmt, sondern aufgrund „logischer und fachlicher Überlegungen“ [12].

Die *Konstruktvaliditäts*-Prüfung der deutschen Version des NCIQ basiert auf kürzlich veröffentlichen Daten von Plath et al. [45]. Die Ergebnisse werden hier nur ausschnittsweise wiedergegeben. Sie wurden aber im Rahmen dieser Studie erneut mittels einer Hauptkomponentenanalyse (Principal Component Analysis, PCA) und der Funktionen der libraries psych, nFactor und FactoMineR der Software *R *reevaluiert. Die PCA dient dazu, umfangreiche Datensätze zu strukturieren, zu vereinfachen und zu veranschaulichen, indem eine Vielzahl statistischer Variablen durch eine geringe Zahl möglichst aussagekräftiger Hauptkomponenten zusammengefasst werden [47]. Zur Bestimmung der Anzahl der Hauptkomponenten wurde eine PCA durchgeführt, die sowohl grafische (Elbow-Plot) als auch numerische Verfahren (Kaiser-Guttmann-Kriterium, Parallelanalyse, Resampling-Methode) ermöglichen [6, 30]. Als Entscheidungskriterium wurde zuletzt das Ergebnis der Parallelanalyse verwendet. Es wurde sich für eine Anzahl von Hauptkomponenten entschieden, deren Eigenwert größer oder gleich dem durchschnittlichen Eigenwert war. Zur Bestätigung dieser Entscheidung wurde noch das Ergebnis der Resampling-Methode herangezogen, um zu prüfen, ob auf deren Basis eine andere Entscheidung getroffen werden würde. In keiner der drei Kohorten, für die eine PCA durchgeführt wurde, unterschieden sich diese beiden Entscheidungskriterien in ihrer Ausprägung.

Im Unterschied zur Originalarbeit von Hinderink et al. [25] wurde die *Übereinstimmungsvalidität* der deutschen Version des NCIQ mittels Korrelationsanalyse (Pearson-Korrelationskoeffzient r) unter Einschluss von zwei krankheitsspezifischen und nicht von generischen Fragebögen untersucht. Hierzu wurden die drei NCIQ-Hauptdomänenwerte auf Übereinstimmung mit den Ergebnissen der anderen krankheitsspezifischen Fragebögen getestet. Im Speziellen wurde die physische Hauptdomäne des NCIQ einerseits mit den vier APHAB-Subdomänen und andererseits mit der HPS-Dimension der „Hörbeeinträchtigung“ auf Korrelation getestet. Bei der psychischen Hauptdomäne des NCIQ erfolgte eine Korrelationsprüfung mit der HPS-Dimension „Selbstwertgefühl“. Die soziale NCIQ-Hauptdomäne wurde auf Korrelation mit der HPS-Dimension „Sozialintegrationsniveau“ geprüft. Gemäß der statistischen Beratung durch das GESIS-Leibniz-Institut sollten im Rahmen der Übereinstimmungsvaliditätsprüfung ausschließlich Fragebögen verwendet werden, die entsprechend vergleichbare krankheitsspezifische Aspekte wie der NCIQ abfragen. Folglich wurde auf eine Korrelationsprüfung mit generischen Fragebögen verzichtet, da sie weniger empfindlich auf krankheitsspezifische Veränderungen des Gesundheitsstatus reagieren. Krankheitsspezifische Instrumente hingegen zielen darauf ab, Informationen über krankheitsspezifische Gesundheitsprobleme zu sammeln [19] und reagieren tendenziell empfindlicher auf behandlungsbedingte Veränderungen [56].

In Anlehnung an die Originalveröffentlichung von Hinderink et al. [25] wurden die Ergebnisse der CI-Gruppe präoperativ ebenso mit den Antworten der ertaubten unbehandelten Kontrollgruppe mittels t‑Test für unverbundene Stichproben verglichen. Um zu beurteilen, ob der deutschsprachige NCIQ auch zwischen ungleichen Gruppen unterscheidet, wurde zudem ein Vergleich mit den 3M- und 6M-CI-Gruppen durchgeführt. Obgleich nicht von Hinderink et al. in der Originalveröffentlichung [25] explizit als *differenzielle Validität (Synonym: diskriminative Validität)* benannt [31], beschreibt der Vergleich ungleicher und gleicher Gruppen miteinander genau dieses Verfahren und wird entsprechend in unserer Arbeit als diskriminative oder differenzielle Validität aufgeführt. Das Ziel war es, aufzuzeigen, dass sich mithilfe der deutschen Version des NCIQ kein Unterschied zwischen einer unbehandelten gehörlosen Kontrollgruppe und einer ebenso erkrankten prätherapeutischen Patientengruppe nachweisen lässt, währenddessen Unterschiede nach einer Cochleaimplantation durchaus existieren.

### Statistische Gütekriterien

Zur Beurteilung, ob mittels der deutschen Version des NCIQ tatsächlich Unterschiede (Effekte) infolge der Cochleaimplantation erkannt werden können, wurde die Trennschärfe bzw. die Sensitivität berechnet. Die Trennschärfe gibt an, mit welcher Wahrscheinlichkeit ein statistischer Test die abzulehnende Nullhypothese korrekt zurückweist und die Alternativhypothese annimmt [16]. Zur Bestimmung der praktischen Relevanz von statistisch signifikanten Ergebnissen wurde die Effektstärke mittels der „standardized mean difference“ (SMD), auch als Cohen d bezeichnet, berechnet. Cohen d wird als Effektstärkemaß für den Vergleich von zwei Mittelwerten verwendet. Berechnet wird Cohen d aus der Differenz der beiden Mittelwerte geteilt durch die gepoolte Standardabweichung („Gesamt-Standardabweichung“) beider Gruppen. Ein Wert kleiner als 0,5 gilt als kleiner Effekt, ein Wert zwischen 0,5 und 0,8 zählt als mittlerer Effekt und Werte darüber als großer Effekt [1, 8]. In unserer Studie wurde die Effektstärke möglicher Änderungen des NCIQ im zeitlichen Verlauf (präoperativ zu 3 Monate postoperativ) berechnet.

Darüber hinaus wurde die ROC-Kurve („Receiver Operating Characteristic“; auch Grenzwertoptimierungskurve genannt) zum visuellen Vergleich der Sensitivität und Spezifität bestimmt. Dies wurde sowohl für den NCIQ-Gesamtscore als auch für die drei Hauptdomänen durchgeführt. Die ROC-Kurve stellt visuell die Abhängigkeit der Effizienz mit der Fehlerrate für verschiedene Parameterwerte dar. Als weiteres Gütekriterium wurde die Fläche unter der ROC-Kurve („Area Under the Curve“, AUC) berechnet. Dieser Wert kann zwischen 0 und 1 liegen. Entsprechend eines Zufallsprozesses bedeuten Werte nahe der Diagonalen eine gleiche Treffer- und Falsch-positiv-Quote. Demnach ist der „schlechteste“ Wert nahe 0,5. Ein Wert nahe 1 (bzw. zwischen 0,5 und 1) kann demnach als gut gedeutet werden. Werte nahe Null signalisieren, dass die Klassifikation in umgekehrter Reihenfolge besser wäre [17, 18].

### Statistische Analyse

Die statistische Analyse wurde von einem der Autoren als zertifizierten Experten für Umfrageanalysen des GESIS-Leibniz-Instituts für Sozialwissenschaften durchgeführt. Die Daten wurden mit der Statistiksoftware R (Version 4.0.2) analysiert [53]. Metrische Variablen wurden als Mittelwert ± Standardabweichung dargestellt. Als metrische Variablen wurden dabei die Werte des NCIQ, des APHAB und des HPS sowie Variablen des Alters (wenn nicht anders gekennzeichnet) verstanden. Für binäre (bspw. Geschlecht oder ein-/beidseitig ertaubt) oder kategoriale (bspw. Schulbildung oder Familienstand) Variablen wurde die relative Verteilung berichtet. Unterschiede in den Fragebogenwerten (bspw. NCIQ-Gesamt- und Hauptdomänenwerte) zwischen den Gruppen wurden unter Verwendung eines ungepaarten t‑Tests bestimmt. Die t‑Test-Ergebnisse wurden mit dem t‑Wert, der Anzahl der zugehörigen Freiheitsgrade („degrees of freedom“, df) und dem aus t‑Wert und Verteilungsfunktion abgeleiteten *p*-Wert angegeben. Diese im Ergebnisteil (für t‑Tests) aufgeführten *p*-Werte wurden anschließend nochmals gemäß der Methode nach Holm [28] adjustiert, wenn eine Korrektur für multiples Testen erforderlich war. Alle (bei Erforderlichkeit adjustierten) *p*-Werte kleiner gleich 0,05 wurden als statistisch signifikant angesehen. Um die Korrelation zwischen den verschiedenen Fragebogeninventaren und den verschiedenen Studiengruppen zu bestimmen, wurde der Pearson-Korrelationstest unter Angabe des Korrelationskoeffizienten r zwischen den einzelnen Werten und Zeitpunkten durchgeführt. Die Testentscheidung erfolgte ebenfalls anhand des (adjustierten) *p*-Werts.

Zusätzlich wurden für alle drei Messzeitpunkte der CI-Gruppe präoperativ Varianzanalysen (ANOVA) zur Bestimmung des Einflusses einzelner Variablen auf die jeweiligen NCIQ-Werte berechnet. Hierbei sollte geprüft werden, ob und welche Charakteristika neben der Taubheit die Höhe des NCIQ-Werts zusätzlich beeinflussen könnten.

Für die CI-Gruppe präoperativ wurden dafür drei lineare Modelle miteinander verglichen:

- Das erste Modell verwendet als Prädiktor lediglich die Variable „Taubheit“ (einseitig oder beidseitig ertaubt).
- Das zweite Modell erweitert das erste Modell um die Variablen „mit/ohne Hörgerät“ (ja oder nein) sowie „Ertaubungsdauer in Monaten“.
- Das dritte Modell erweitert Modell 2 um die soziodemografischen Variablen „Geschlecht“, „Familienstand“ und „Alter“.

Da einige der hier aufgeführten Variablen im Zuge der Messung der Kontrollgruppe nicht erhoben wurden, konnten diese drei Modelle nicht für die Kontrollgruppe gebildet werden. Anstelle dessen wurden die folgenden drei Modelle miteinander verglichen:

- Modell 1_c: Seite des Cochleaimplantats,
- Modell 2_c: Seite des Cochleaimplantats sowie Alter bei Operation,
- Modell 3_c: Seite des Cochleaimplantats, Alter bei Operation sowie Geschlecht.

Die Auswahl und Entscheidung für eines der Modelle erfolgte dabei anhand der jeweiligen *p*-Werte unter der Verwendung der F‑Statistiken unter der Nullhypothese, dass die zusätzlichen Variablen im Vergleich zum ersten Modell (gemeinsam) Koeffizienten gleich 0 aufweisen (*Pr(>* *F)*). Zusätzlich wurden Werte für Cronbach‑α zur internen Konsistenzprüfung berechnet. Eine Hauptkomponentenanalyse zur Überprüfung der Anzahl der Einzelkomponenten des NCIQ erfolgte bereits in einer vorherigen Auseinandersetzung mit dem zugrunde liegenden Patientenkollektiv [45], welche hier nochmals repliziert wurde. Darüber hinaus wurden die SMD zur Messung der Sensitivität verwendet. Die einzelnen Verfahren werden im Zuge der Ergebnisanalyse nochmals genauer benannt.

## Ergebnisse

### Studiengruppen

Zu Beginn der Untersuchung wurden 100 taube Patienten (54 % Frauen/46 % Männer) in die Studie eingeschlossen (CI-Gruppe präoperativ). Das durchschnittliche Alter lag zum Zeitpunkt der geplanten Operation bei 55,3 ± 16,9 Jahren (Altersspanne: 18,7–87,4 Jahre). Dabei klagten 57 % der Patienten über eine einseitige Ertaubung, 43 % über eine beidseitige Ertaubung. Die durchschnittliche Dauer der Ertaubung betrug 213,2 ± 203,6 Monate (Spanne: 2–659 Monate).

Im weiteren Verlauf konnten drei Monate nach Cochleaimplantation die Fragebögen von 85 Teilnehmern (55,3 % Frauen/44,7 % Männer) mit einem mittleren Alter von 56,9 ± 16,9 Jahren (Altersspanne: 19,5–87,4) in die Auswertung miteinbezogen werden (CI-3M-Gruppe). Nach sechs Monaten reduzierte sich die Anzahl auf 65 teilnehmende Probanden (52,3 % Frauen/47,7 % Männer) mit einem mittleren Alter von 57,0 ± 15,5 Jahren bei einer Altersspanne von 19,5–87,4 (CI-6M-Gruppe). Zur besseren Vergleichbarkeit eines ebenso erkrankten, aber noch unbehandelten Kollektivs wurden bei der Kontrollgruppe (*n* = 54) nur die präoperativen Daten in diese Auswertung eingeschlossen. Das durchschnittliche Alter der Kontrollgruppe betrug 59,2 ± 14,4 Jahre (Altersspanne: 19–71 Jahre), 56 % waren weiblich.

**Figure 2:**
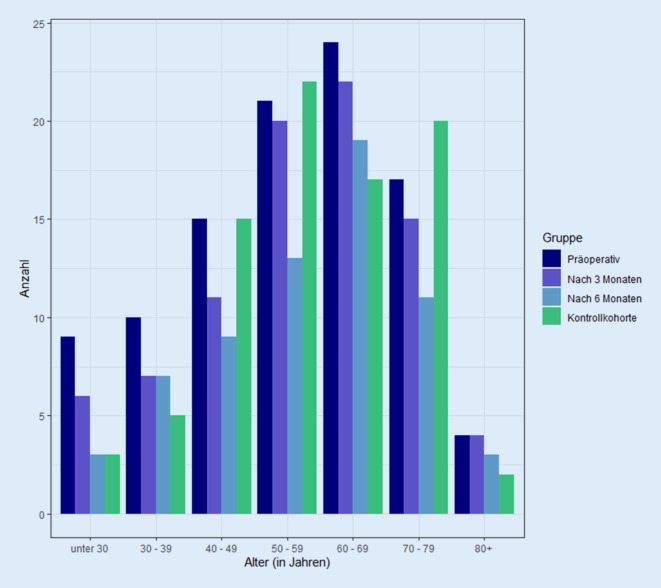

Die Altersverteilung der hier untersuchten Studiengruppen ist in der Abb. 2 dargestellt.

Der Hauptfokus dieser Studie lag in der Ergebnisanalyse der präoperativ gewonnenen Datensätze, welche zur Validität- und Reliabilitätsprüfung mit denen der postoperativen Daten und denen der unbehandelten Kontrollgruppe verglichen wurden. Die Ergebnisse des NCIQ-Gesamt-, der Haupt- und Subdomänenwerte der jeweiligen Studiengruppe sind in Tab. 1 als Übersicht dargestellt.

**Table 1:** 

Nijmegen Cochlear Implant Questionnaire	Kontrollgruppe (*n* = 54)	CI-Gruppe präoperativ (*n* = 100)	CI-3M-Gruppe (*n* = 85)	CI-6M-Gruppe (*n* = 65)
*Gesamtwert*	52,72 ± 15,75	49,91 ± 17,85	64,41 ± 15,31	67,96 ± 13,64
*Physische Komponente*	57,62 ± 17,17	56,37 ± 20,48	70,55 ± 16,35	74,49 ± 13,53
Elementare Geräuschwahrnehmung	49,34 ± 21,31	48,11 ± 22,28	67,26 ± 19,34	72,88 ± 13,99
Fortgeschrittene Geräuschwahrnehmung	54,34 ± 20,70	50,68 ± 24,37	64,01 ± 19,75	68,69 ± 18,54
Sprachproduktion	69,19 ± 18,31	70,33 ± 22,62	80,39 ± 17,95	81,89 ± 17,50
*Psychologische Komponente*	50,61 ± 16,13	43,82 ± 18,38	55,89 ± 17,56	59,45 ± 15,87
Selbstwertgefühl	50,61 ± 16,13	43,82 ± 18,38	55,89 ± 17,56	59,45 ± 15,87
*Soziale Komponente*	46,34 ± 18,23	43,61 ± 17,33	59,20 ± 17,17	62,42 ± 16,51
Aktivitäten	44,62 ± 19,35	42,05 ± 18,86	57,43 ± 17,77	60,16 ± 18,31
Soziale Interaktionen	48,06 ± 18,93	45,16 ± 17,61	60,96 ± 19,20	64,68 ± 16,48

### Modellvergleiche zur Erklärung des NCIQ-Werts anhand von Varianzanalysen

Zur näheren Untersuchung des Einflusses unterschiedlicher Variablen auf die Höhe des NCIQ-Werts erfolgte zu unterschiedlichen Messzeitpunkten eine Varianzanalyse (ANOVA) der drei zuvor beschriebenen Modelle, die neben medizinischen auch soziodemografische Parameter zur Erklärung der jeweiligen NCIQ-(Sub‑)Werte beinhaltete. Anhand dieser Testung zeigte sich (zu unterschiedlichen Zeitpunkten), dass die Hinzunahme weiterer Prädiktoren wie „Ertaubungsdauer“ (Modell 2) oder soziodemografischer Variablen wie „Geschlecht“, „Familienstand“ und „Alter“ (Modell 3) den Erklärungsgehalt der Schätzung durch das „Minimal-Modell“ nicht weiter erhöht (Modell 2 vor CI: Pr(> F) = 0,33; Modell 3 vor CI: Pr(> F) = 0,32; Modell 2 nach CI (3 Monate): Pr(> F) = 0,71; Modell 3 nach CI (3 Monate): Pr(> F) = 0,32; Modell 2 nach CI (6 Monate): Pr(> F) = 0,15; Modell 3 nach CI (6 Monate): Pr(> F) = 0,62). Nach den hier durchgeführten Analysen bewährte sich das „Minimal-Modell“ (Modell 1), welches lediglich die „Seite der Ertaubung“ als Prädiktor verwendet, gegenüber den beiden komplexeren Modellen. Jedoch gilt anzumerken, dass der Anteil der erklärten Varianz, gemessen durch das adjustierte Bestimmtheitsmaß, zu keinem der beiden betrachteten Zeitpunkte und in keinem der drei Modelle einen großen Anteil der Variation der NCIQ-Werte erklärt. So lagen diese zwischen 9 % bei Modell 1 und 11 % bei Modell 3.

Auch für die Kontrollkohorte wurde ein entsprechender Vergleich angestrebt. Wie zuvor beschrieben, war hier jedoch eine direkte Adaption des Modells, das zu den drei Messzeitpunkten für die klinische Kohorte verwendet wurde, nicht möglich. Die Ergebnisse beziehen sich daher auf die Modellbeschreibung der Kontrollgruppe, die bereits aufgeführt wurde, und sind mit den vorherigen Ergebnissen nicht direkt vergleichbar. Jedoch zeigt sich auch hier, dass die Hinzunahme weiterer Variablen fernab der Seite des Cochleaimplantats keinen signifikanten Zugewinn an erklärter Varianz liefert (Modell 2_c: Pr(> F) = 0,25; Modell 3_c: Pr(> F) = 0,66).

### Reliabilitätsprüfung

#### Interne Konsistenz

Unter Berücksichtigung der drei Hauptdomänen (physisch, psychisch und sozial) wies der NCIQ-Gesamtwert der CI-Gruppe präoperativ mit einem Cronbach‑α von 0,89 und einem Konfidenzintervall von 0,85–0,93 eine gute interne Konsistenz auf. Alle drei Hauptdomänen zeigten einzeln betrachtet eine gute interne Konsistenz mit Werten von mindestens 0,79 (physisch: 0,89, psychisch: 0,85, sozial: 0,79) auf. Unter Einbeziehung der sechs NCIQ-Subdomänen betrug der Gesamt-Cronbach-α-Wert 0,91 (Konfidenzintervall: 0,88–0,94), die Cronbach-α-Werte der sechs Subdomänen waren jeweils $\geq$0,87 (Sound Perception Basic: 0,9, Sound Perception Advanced: 0,87, Speech Production: 0,89, Self-Esteem: 0,88, Activity: 0,87 und Social Interaction: 0,88). Sowohl bei der Kontrollgruppe als auch bei den CI-3M- und CI-6M-Gruppen waren alle berechneten α‑Werte sowohl unter der einfachen Berücksichtigung der drei Hauptdomänen als auch unter der Verwendung der sechs Subdomänen $\geq$0,7.

#### Treatment-Effekte und Retest-Reliabilität

Zur Überprüfung, ob sich nach einer Cochleaimplantation eine Veränderung der einzelnen NCIQ-Werte einstellt (Treatment-Effekt), wurden t‑Tests für den NCIQ-Gesamt- sowie für die drei Hauptdomänenwerte durchgeführt.

Die Ergebnisse der CI-Gruppe präoperativ unterschieden sich im NCIQ-Gesamtwert und in den drei Hauptdomänen hoch signifikant von den Resultaten der CI-3M-Gruppe: CI-Träger wiesen drei Monate nach Cochleaimplantation gegenüber der präoperativ untersuchten Studiengruppe sowohl einen verbesserten NCIQ-Gesamt- (64,41 vs. 49,91; *p* < 0,001; t = −5,95; df = 182,98), physischen (70,55 vs. 56,37; *p* < 0,001; t = −5,23; df = 182,33), sozialen (59,2 vs. 43,61; *p* < 0,001; t = −6,13; df = 178,73) als auch einen verbesserten psychischen Wert (55,89 vs. 43,82; *p* < 0,001; t = −4,56; df = 180,47) auf.

Zwischen den CI-3M- und CI-6M-Gruppen konnten mittels t‑Tests keine statistisch signifikanten Unterschiede der NCIQ-Gesamtwerte festgestellt werden. Weder für den Gesamtwert (CI-3M-Gruppe: 64,41 vs. CI-6M-Gruppe: 67,96; *p* = 0,546; t = −1,5; df = 144,5), noch für die physische (CI-3M-Gruppe: 70,55 vs. CI-6M-Gruppe: 74,49; *p* = 0,438; t = −1,61; df = 147,05), soziale (CI-3M-Gruppe: 59,2 vs. CI-6M-Gruppe: 62,42; *p* = 0,984; t = −1,16; df = 140,46) oder psychische (CI-3M-Gruppe: 55,89 vs. CI-6M-Gruppe: 59,45; *p* = 0,783; t = −1,3; df = 143,86) Hauptdomäne lassen sich signifikante Unterschiede feststellen.

In den anschließenden Korrelationsanalysen zur Retest-Reliabilitätsprüfung ergaben sich zwischen den CI-3M- und 6M-Gruppen statistisch hoch signifikante lineare Zusammenhänge der NCIQ-Gesamt- und der Haupt- und Subdomänenwerte (NCIQ-Gesamtwert: r = 0,81; *p* < 0,001; t = 11,03; df = 63; Physischer Wert: r = 0,79; *p* < 0,001; t = 10,28; df = 63) (Speech Production: r = 0,80; *p* < 0,001; t = 10,437; df = 63/Sound Perception Advanced: r = 0,70; *p* < 0,001; t = 7,78; df = 63/Sound Perception Basic: r = 0,74; *p* < 0,001; t = 8,62; df = 63); sozialer Wert: r = 0,79; *p* < 0,001; t = 10,25; df = 63 (Activity: r = 0,74; *p* < 0,001; t = 8,79; df = 63/Social Interaction: r = 0,80; *p* < 0,001; t = 10,56; df = 63); psychischer Wert/Self-Esteem: r = 0,81; *p* < 0,001; t = 11,06; df = 63.

Die vorherigen t‑Tests vor und 3 Monate nach Cochleaimplantation zeigen, dass sich hier durchaus ein Treatment-Effekt in die erwartete Richtung zeigt. Angenommen, dass der NCIQ korrekt die QoL misst, lässt sich demnach festhalten, dass diese nach Cochleaimplantation steigt. Dieser Unterschied zeigt sich beim Vergleich der CI-3M- und 6M-Gruppen nicht. Es kann davon ausgegangen werden, dass der nach 3 Monaten nach Cochleaimplantation gemessene erhöhte Wert auch nach 6 Monaten (vergleichsweise) unverändert erhöht bleibt. Folglich lässt sich mittels der Korrelationsanalyse ein zufriedenstellendes Ergebnis der Retest-Reliabilitätsprüfung nachweisen, da ein und dasselbe Testinstrument (hier: deutsche Version des NCIQ) zu zwei verschiedenen Messzeitpunkten (3 und 6 Monate postoperativ) vergleichbare NCIQ-Werte aufweist.

### Messung der Sensitivität, Spezifität und Effektstärke

Ob sich mittels der deutschen Version des NCIQ tatsächlich eine subjektive Veränderung der Lebensqualität infolge einer Cochleaimplantation messen lässt, wurde durch den Einsatz der SMD und der Analyse der ROC-Kurve untersucht. Es waren sowohl beim NCIQ-Gesamtwert als auch bei den drei Hauptdomänen zum Messzeitpunkt drei Monate nach Cochleaimplantation in Bezug zum präoperativen Status mittlere (≥ 0,5 und < 0,8) bis große Effekte (≥ 0,8), gemessen an der SMD, nachweisbar (SMD NCIQ-Gesamtwert von 0,87; SMD NCIQ-physisch von 0,76; SMD NCIQ-psychisch von 0,67; SMD NCIQ-sozial von 0,90).

**Figure 3:**
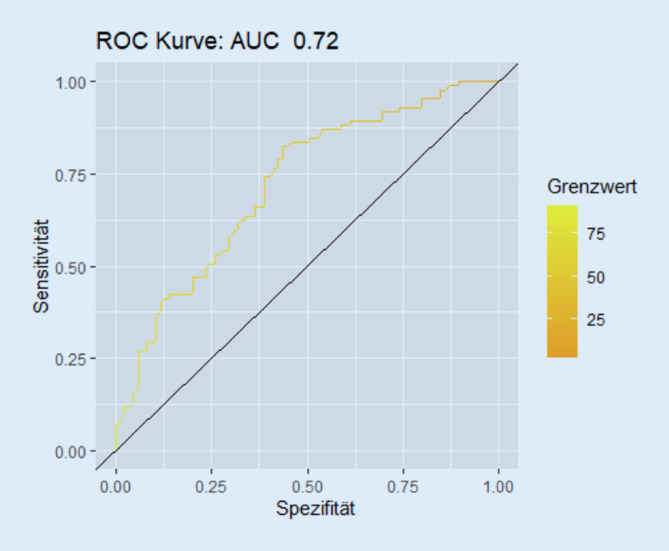

Die Fläche unter der ROC-Kurve (AUC) beträgt 0,72 für den NCIQ-Gesamtwert der CI-Gruppe präoperativ, was darauf hindeutet, dass die Klassifikation mittels NCIQ oftmals korrekt erfolgt (Abb. 3). Für die physische Hauptdomäne des NCIQ ergibt sich eine AUC von 0,69 (Sound Perception Basic: 0,75/Sound Perception Advanced: 0,64/Speech Production: 0,62), für die psychische Hauptdomäne mit Erhebung des Selbstwertgefühls eine AUC von 0,68 und für die soziale Hauptdomäne eine AUC von 0,73 (Activity: 0,72; Social Interaction: 0,73).

### Validitätsprüfung

#### Äquivalenz der Studien- und Kontrollstichproben im Rahmen der differenziellen Validitätsprüfung

Die Ergebnisse des NCIQ-Gesamtwerts und der drei Hauptdomänen der CI-Gruppe präoperativ wurden mittels t‑Tests für unabhängige Stichproben mit denen der Kontrollgruppe verglichen. Der NCIQ-Gesamtwert beider Gruppen unterschied sich statistisch nicht signifikant voneinander (Kontrolle: 52,72 vs. CI-Gruppe präoperativ: 49,91; *p* = 1; t = 1,01; df = 120,87). Weder im physischen (Kontrolle: 57,62 vs. CI-Gruppe präoperativ: 56,37; *p* = 1; t = 0,40; df = 125,94), sozialen (Kontrolle: 46,34 vs. CI-Gruppe präoperativ: 43,61; *p* = 1; t = 0,90; df = 104,05) noch im psychischen NCIQ-Hauptdomänenwert unterschieden sich beide Gruppen voneinander (Kontrolle: 50,61 vs. CI-Gruppe präoperativ: 43,82; *p* = 0,08; t = 2,37; df = 121,43).

Beim Vergleich der Kontrollgruppe mit der CI-3M-Gruppe konnten in allen NCIQ-Werten – mit Ausnahme der psychischen Hauptdomäne – statistisch signifikante Unterschiede festgestellt werden (NCIQ-Gesamtwert der Kontrolle: 52,72 vs. CI-3M-Gruppe: 64,41; *p* < 0,001; t = −4,31; df = 110,56; Physischer Wert der Kontrolle: 57,62 vs. CI-3M-Gruppe: 70,55; *p* < 0,01; t = −4,41; df = 108,92; sozialer Wert der Kontrolle: 46,34 vs. CI-3M-Gruppe: 59,2; *p* < 0,001; t = −4,14; df = 107,93; psychischer Wert der Kontrolle: 50,61 vs. CI-3M-Gruppe: 55,89; *p* > 0,05; t = −1,82; df = 119,99).

#### Übereinstimmungsvalidität

**Figure 4:**
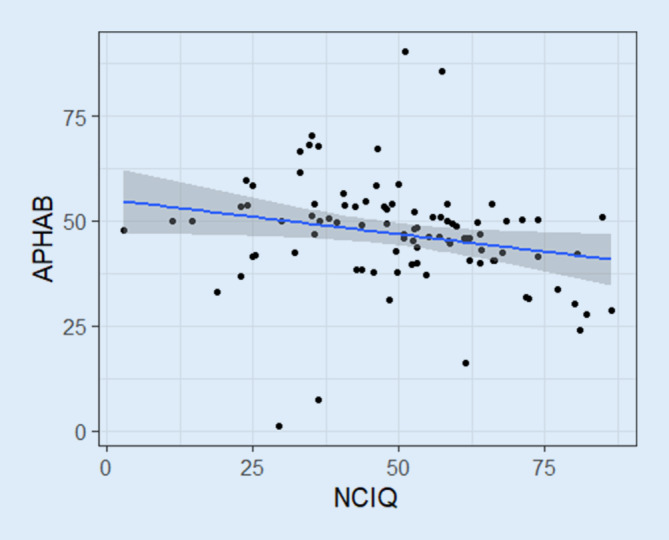

Bei den Untersuchungen der CI-Gruppe präoperativ ergab sich, entsprechend der unterschiedlichen Polung der Fragebögen (NCIQ: hoher Ergebniswert bedeutet Funktionalität, APHAB: hoher Ergebniswert bedeutet Handicap), eine statistisch signifikante schwache, negative Korrelation zwischen dem NCIQ- und dem APHAB-Gesamtwert mit einem Korrelationskoeffizienten von −0,22 und einem Konfidenzintervall von −0,41 bis −0,02 (*p* = 0,04; t = −2,13; df = 90; Abb. 4). Die Korrelationsanalyse der NCIQ-Hauptdomänen zeigte eine negative Korrelation zwischen dem physischen NCIQ-Wert und dem „Ease-of-Communciation(EC)-APHAB-Wert“ (r = −0,48; *p* < 0,001; t = −5,25; df = 90). Es bestand keine Korrelation zwischen dem physischen NCIQ-Wert und dem „Reverberation(RV)-APHAB-Wert“ (r = −0,1; *p* = 1; t = −0,93; df = 90), dem „Background-Noise(BN)-APHAB-Wert“ (r = 0,14; *p* = 0,81; t = 1,29; df = 89) sowie mit dem „Aversiveness-of-Sound(AV)-APHAB-Wert“ (r = 0,21; *p* = 0,17; t = 2,05; df = 90).

Im Rahmen der Prüfung auf Übereinstimmung zwischen dem NCIQ und dem HPS wurden jeweils die 3‑Monats-Werte herangezogen, da der HPS nur postoperativ angewendet wird. Hierbei zeigte sich infolge einer Cochleaimplantation, dass der NCIQ- und der HPS-Gesamtwert statistisch signifikant miteinander korrelierten (r = 0,3; *p* = 0,01; t = 2,82; df = 83). Der physische NCIQ-Wert korrelierte nicht signifikant mit der HPS-Hörbeeinträchtigungs-Dimension (r = 0,09; *p* = 1; t = 0,85; df = 83). Der psychische NCIQ-Wert korrelierte jedoch positiv, hoch statistisch signifikant mit dem HPS-Selbstwertgefühl (r = 0,42; *p* < 0,001; t = 4,33; df = 83), wohingegen der soziale NCIQ-Wert negativ, schwach statistisch signifikant mit dem Sozialintegrationsniveau des HPS bei einem Korrelationskoeffizienten von (r = −0,24; *p* = 0,03; t = −2,25; df = 83) korrelierte.

#### Inhalts- und Konstruktvalidität

Zur Überprüfung der von Hinderink et al. [25] entwickelten dreigegliederten Struktur der englischen Version des NCIQ wurde die Hauptkomponentenanalyse erneut an den Ergebnissen der CI-Gruppe präoperativ durchgeführt und lieferte übereinstimmende Ergebnisse mit den kürzlich veröffentlichen Daten von Plath et al. [45], welche ein zu empfehlendes Dreikomponentenmodell mit einer kumulierten Varianz von 45 % vorschlugen.

## Diskussion

Subjektive Bewertungen der gesundheitsbezogenen Lebensqualität nehmen einen zunehmend größeren Stellenwert in der Ergebnisanalyse und Qualitätskontrolle in der Medizin ein [2]. Zu den „Patient-Reported Outcomes“ der HRQoL gehören sowohl generische als auch krankheitsspezifische Fragebögen, wobei krankheitsspezifische Fragebögen gegenüber der generischen Fragebögen tendenziell empfindlicher auf behandlungsbedingte Veränderungen reagieren [56]. Der Nijmegen Cochlear Implant Questionnaire (NCIQ) fand als krankheitsspezifischer First-Line-Fragebogen zur Erhebung der Lebensqualität bei Patienten vor und nach einer Cochleaimplantation bereits häufig Verwendung [5, 21, 22, 27, 45, 46] und ist zudem fester Bestandteil der neu überarbeiteten S2k-Leitline (AWMF-Register-Nr. 017/071AWMF-Register-Nr. 017/071; Titel: Cochlea-Implantat Versorgung). Die Zielstellung dieser Studie bestand in der Validierung und Reliabilitätsprüfung der deutschen Übersetzung des NCIQ.

Im Rahmen der Prüfung auf Zuverlässigkeit, ob die deutsche Version des NCIQ tatsächlich ein Merkmal ohne Messfehler misst, wurde die *interne Konsistenz *untersucht. Hierbei konnte sowohl für den NCIQ-Gesamt- als auch für die Haupt- und Subdomänenwerte eine gute interne Konsistenz mit Cronbach-α-Werten größer 0,7 festgestellt werden. Unsere Ergebnisse sind folglich mit den Daten der englischen Version von Hinderink et al. [25], welche interne Konsistenzen über 0,7 (geringster Cronbach‑α von 0,73 für Speech Production/höchster Cronbach‑α von 0,89 für Activity) zeigten, und ebenso mit der spanischen Version des NCIQ von Sanchez-Cuadrados et al. [50] mit internen Konsistenzen von über 0,7 (geringster Cronbach‑α von 0,65 für Social Interaction/höchster Cronbach‑α von 0,89 für Activity) vergleichbar. Unsere Cronbach-α-Subdomänenwerte fielen jedoch, gegensätzlich zu den oben genannten Studien, allesamt einheitlich hoch aus (> 0,87), was für eine hohe interne Konsistenz und eine gute Reliabilität der deutschen Version des NCIQ spricht.

Als weiteres Maß der Reliabilitätsprüfung wurde die *Retest-Reliabilität* bestimmt. Im Einklang mit der aktuellen Literatur konnte in unserer Studie initial eine statistisch signifikante Verbesserung der hörspezifischen gesundheitsbezogenen Lebensqualität mittels Messung des deutschsprachigen NCIQ [9, 13, 25, 27] gezeigt werden, woraufhin eine Stabilität der NCIQ-Werte drei und sechs Monate nach Cochleaimplantation folgte [40, 45]. Bei näherer Betrachtung unserer Ergebnisse fiel jedoch auf, dass die CI-6M-Gruppe minimal höhere, statistisch nicht signifikante Haupt- und Subdomänenwerte als die CI-3M-Gruppe aufwies, was möglicherweise auf die große interindividuelle Variabilität der Werte, die unterschiedliche Patientenpower (85 vs. 65) oder den später eintretenden Behandlungserfolg nach Cochleaimplantation zurückzuführen ist. Dennoch signalisiert diese weitestgehend vorhandene Stabilität der postoperativ gemessenen NCIQ-Werte, dass sich das zu messende Merkmal selbst nicht statistisch signifikant verändert hat und dass folglich die deutsche Version des NCIQ in Verbindung mit der Interpretation der Korrelationsanalysen der CI-3M- und CI-6M-Gruppe reliabel misst. Bei der Retest-Reliabilität ist weiter zu beachten, dass die ermittelte Korrelation in Abhängigkeit vom Zeitintervall zwischen beiden Testungen variieren kann, da je nach gewähltem Zeitabstand eine Vielzahl von Einflüssen auf die Messungen, solche wie Übungs- und Erinnerungseffekte oder ein sich veränderndes Persönlichkeitsmerkmal, denkbar sind und sich reliabilitätsverändernd auswirken können [38]. Mögliche, das Ergebnis verfälschende Veränderungen der gemessenen Testwerte konnten in unserer Studie jedoch mithilfe der Sensitivitätsanalysen ausgeschlossen werden, die eine gute Messqualität der deutschsprachigen NCIQ widerspiegelten. Bei der Sensitivitätsprüfung konnten sowohl beim NCIQ-Gesamtwert als auch bei den drei Hauptdomänenwerten zum Messzeitpunkt vor und drei Monate nach Cochleaimplantation mittlere bis große Effektstärken nachgewiesen werden. Die Fläche unter der ROC-Kurve wies für den NCIQ-Gesamtwert oftmals auf eine korrekte Klassifikation der QoL mittels NCIQ hin.

Das Gütekriterium der Validität befasst sich mit der inhaltlichen Übereinstimmung der vom deutschsprachigen NCIQ gemessenen Ergebnisse und wurde in unserer Studie mittels der Inhalts‑, Übereinstimmungs-, differenziellen Validität sowie mittels der Reevaluation kürzlich veröffentlichter Daten zur Konstruktvalidität untersucht.

Dass die deutsche Version des NCIQ das zu messende Merkmal korrekt erfasst und somit *inhaltsvalide* ist, können wir durch die übereinstimmenden Ergebnisse mit weiteren Vorarbeiten [5, 20, 22, 25, 27, 44, 45] bestätigen, die allesamt eine verbesserte Lebensqualität mittels des deutschsprachigen NCIQ infolge einer Cochleaimplantation zeigten. Kliniker und politische Entscheidungsträger haben mittlerweile erkannt, dass Veränderungen der Lebensqualität oder des Gesundheitszustands eines Patienten das primäre Ziel medizinischer Interventionen sind. Abgesehen von der Verbesserung der Hör- und Sprachproduktion werden infolge der CI-Versorgung das Selbstwertgefühl, die täglichen Aktivitäten und das soziale Leben positiv beeinflusst [25]. Chinesische [15] und italienische [43] Studien validierten den NCIQ ausschließlich auf Inhaltsvalidität mithilfe einer Literaturrecherche.

Im Rahmen der Prüfung der *differenziellen Validität* konnte festgestellt werden, dass der NCIQ-Fragebogen zwischen prä- und postoperativen Werten der klinischen Gruppen und Werten einer noch unbehandelten gehörlosen Kontrollgruppe diskriminierte. Dabei fielen erwartungsgemäß der NCIQ-Gesamtwert sowie die physischen, sozialen und psychischen NCIQ-Hauptdomänenwerte der Kontroll- und präoperativen Studiengruppe similär aus. In Anlehnung an die Originalveröffentlichung von Hinderink et al. [25] rekrutierten wir eine Kontrollgruppe von 54 postlingual gehörlosen oder hochgradig schwerhörigen Patienten nach ähnlichen soziodemografischen Charakteristika wie unsere CI-Gruppe präoperativ (CI-Gruppe präoperativ: 55,3 ± 16,9 Jahre, Altersspanne: 18,7–87,4 Jahre vs. Kontrollgruppe: 59,2 ± 14,4 Jahre, Altersspanne: 19–71 Jahre; 54 % vs. 56 % weiblich).

Hinderink et al. überprüften die *Übereinstimmungsvalidität *der englischen Version des NCIQ unter Verwendung generischer Lebensqualitätsmessinstrumente [25]. Da generische Fragebögen eine Vielzahl von Aspekten des Gesundheitszustands einer Person erfassen, konzentrieren sie sich auf Zustände, die möglicherweise nicht durch eine Cochleaimplantation beeinflusst werden, und sind daher tendenziell weniger empfindlich [19], um Lebensqualitätsveränderungen und das funktionelle Ergebnis durch Cochleaimplantation zu bewerten. Korrelationsprüfungen mit krankheitsspezifischen Fragebögen wie dem APHAB und dem HPS, welche beide ähnliche Aspekte der drei Hauptdomänen des NCIQ erfassen, scheinen hierbei sinnvoller zu sein. Die parametrische Korrelationsanalyse ergab eine statistisch signifikante Übereinstimmungsvalidität zwischen dem NCIQ- und dem APHAB-Gesamtwert mit negativer Korrelation. Dies scheint plausibel, da die beiden Fragebögen in entgegengesetzter Richtung (NCIQ: hoher Ergebniswert bedeutet Funktionalität, APHAB: hoher Ergebniswert bedeutet Handicap) geordnet sind. Der APHAB ist ein aus dem Bereich der Hörgeräte übernommener Fragebogen. Im APHAB-Fragebogen ist ein ausgeprägtes Hörhandicap mit einer größeren Punktzahl verbunden, währenddessen ein geringeres Hörhandicap mit einer kleineren Punktzahl assoziiert ist [10]. Der APHAB hat einen physisch ausgerichteten Schwerpunkt. Die physische NCIQ-Hauptdomäne wurde entsprechend mit den APHAB-Hauptdomänen auf Korrelation getestet. Hierbei konnte festgestellt werden, dass die physische NCIQ-Hauptdomäne mit dem EC-APHAB-negativ statistisch signifikant korrelierte. Der physische NCIQ-Wert korrelierte nicht signifikant mit dem BN-APHAB, dem AV-APHAB und dem RV-APHAB. Zur Prüfung der sozialen und psychischen Hauptdomänen des NCIQ erfolgte eine Übereinstimmungsanalyse mit dem HPS, obgleich bisher noch keine Validierung der deutschen HPS-Version vorliegt [4]. Mit seinen drei Hördimensionen – Selbstwertgefühl bezüglich des Hörens, Sozialintegrationsniveau und Hörbeeinträchtigung – eignet sich der HPS, welcher in Studien ausschließlich postoperativ eingesetzt wird [37], besser als der APHAB oder der HHIE (Hearing Handicap Inventory for the Elderly) zur Korrelationsprüfung aller drei NCIQ-Hauptdomänen. Es ergaben sich jedoch keine statistisch signifikanten Korrelationen zwischen dem physischen NCIQ-Wert und der HPS-Hörbeeinträchtigungs-Dimension. Von statistischer Signifikanz waren jedoch die Korrelationen zwischen dem sozialen NCIQ-Wert und dem Sozialintegrationsniveau des HPS (negative Korrelation) sowie zwischen der psychischen NCIQ-Hauptdomäne mit der HPS-Selbstwertgefühl-Dimension (positive Korrelation). Die negative Korrelation zwischen den Werten der sozialen Hauptdomäne des NCIQ und dem Sozialintegrationsniveau des HPS scheinen an dieser Stelle kontraintuitiv. Eine mögliche Begründung ist an dieser Stelle schwer zu treffen. Das Ergebnis kann neben einer höheren und unterschiedlichen Variabilität der Index-Werte dieser beiden Domänen auch über die damit verbundene größere erforderliche Power beeinflusst worden sein. Daneben ist es durchaus möglich, dass beide Hauptdomänen grundsätzlich unterschiedliche Gegenstände/Sachverhalte abbilden. Darüber hinaus ist der HPS in deutscher Sprache noch nicht validiert. Dennoch signalisieren diese gemessenen signifikanten Korrelationen zwischen dem HPS und dem NCIQ, dass soziale und psychische Aspekte eine wichtige Rolle im Bereich der gesundheitsbezogenen Lebensqualität nach Cochleaimplantation zu spielen scheinen.

Im Rahmen der NCIQ-Entwicklungs- und Validierungsstudie von Hinderink et al. wurde die *Konstruktvalidität* der englischen Version des NCIQ mittels einer Faktorenanalyse getestet [25]. Auch Sanchez-Cuadrado et al. führten im Rahmen der Validierung des NCIQ in spanischer Sprache eine Faktorenanalyse durch, wobei kein klares strukturelles Muster festzustellen war, da die Mehrzahl der Items auf den gleichen Faktor zuzuordnen waren [50]. Mit dem Ziel, zum einen die etablierte (Haupt‑) Domänenklassifizierung des NCIQ zu überprüfen und zum anderen mögliche Änderungen vorzuschlagen, die dazu beitragen könnten, die Hochdimensionalität des Fragebogens auf wenige Komponenten zu reduzieren, führten wir erneut die Hauptkomponentenanalyse im Rahmen der Konstruktvaliditätstestung durch. In Übereinstimmung mit der ursprünglichen Unterteilung der englischen Version des NCIQ von Hinderink et al. [25] konnte hierbei ebenfalls ein Dreikomponentenmodell des NCIQ mit jedoch unterschiedlicher Zuordnung der Fragen zu der jeweiligen Hauptdomäne festgestellt werden, welche von Plath et al. erstmals als „physische“, „physisch fortgeschrittene“ und „soziopsychologische“ Hauptdomänen bezeichnet wurden [45]. Da inhaltlich aber nichts an den jeweiligen 60 Items des deutschsprachigen NCIQ verändert wurde, sondern in Plath et al. lediglich die übergreifenden Hauptdomänen unterschiedlich betitelt worden sind, hat die neue Zuordnung der Items keine Konsequenz auf den Einsatz der deutschen Version im klinischen Praxisalltag. Zudem blieb der NCIQ-Gesamtwert davon unberührt. Obgleich das Dreikomponentenmodell lediglich eine kumulative Varianz von 45 % erklären konnte, führte die zusätzliche Aufnahme weiterer Faktoren lediglich zu einem geringen zusätzlichen Anstieg erklärter Varianz. Anhand dieser Ergebnisse und der hier durchgeführten Hauptkomponentenanalyse kann also davon ausgegangen werden, dass keine zusätzliche Variation durch die Hinzunahme weiterer Komponenten erfolgt, die die Variation der Faktoren innerhalb der zusätzlichen Komponente (und erneuten Einteilung) übersteigt. Der vergleichsweise geringe Anteil der erklärten Varianz lässt indes vermuten, dass ein Großteil der unerklärten Varianz entweder auf zusätzliche Confounder zurückzuführen ist oder, dass die Stichprobengröße höher hätte sein müssen, um deutlichere Ergebnisse darzulegen. Letzteres hätte sich insgesamt positiv auf die Varianz der Schätzwerte sowie auf die Aussagekraft der einzelnen Tests ausgewirkt.

Die vorliegende Studie weist einige Limitationen auf. Bei der Beurteilung der Lebensqualität werden kognitive Fähigkeiten wie die Reflexion, Introspektion, Informationswahrnehmung, -verarbeitung und -wiedergabe, mit denen funktionelle Einschränkungen vor und nach Cochleaimplantation richtig bewertet werden können, nicht berücksichtigt. Zudem birgt die Selbstauskunft bei „Patient-Reported Outcome Measures“ (PROMs) immer das Risiko einer Antwortverzerrung („response bias“), sodass die erhobenen Daten nicht die zutreffenden „wahren“ Sachverhalte abbilden [42]. Die Antwortverzerrung gilt als ein typisches Methodenproblem von Fragebögen und lässt sich somit nicht gänzlich vermeiden [48]. Weiterhin liegt das mittlere Alter der CI-Gruppe präoperativ bei 55,3 ± 16,9 Jahren, sodass Erfahrungen jüngerer (< 30 Jahre; 9 %) und älterer (> 80 Jahre; 4 %) ertaubter Patienten bei unserer Datenanalyse nur eine geringe Beachtung fanden. Da jüngere Menschen in der Regel aber gut hören und Höreinschränkungen bei älteren Menschen pathophysiologisch häufiger auftreten und gesellschaftlich akzeptiert sind [42], spielen gerade Untersuchungen an einem „mittelalten“ Patientenkollektiv eine zunehmend wichtige Rolle, beispielsweise für Anregungen zur Fortentwicklung von Cochleaimplantat-Technologien. Als weitere Kritik ist die hohe Abbrecherquote von 45 % der CI-Gruppe präoperativ nach 6 Monaten zu nennen, welches zu einem Selektion-Bias führen kann [37] und die hier aufgeführten Ergebnisse möglicherweise beeinflusst hat. So besteht beispielsweise die Möglichkeit, dass tendenziell eher diejenigen, die entweder sehr positive oder sehr negative Erfahrungen nach CI gemacht haben, zur Wiederbefragung bereit sind. Trifft dies zu, so über- oder unterschätzt man die Veränderung der NCIQ-Werte nach CI. Anhand der vorliegenden Daten lassen sich dafür jedoch keine Hinweise finden. Zudem fanden bei der Übereinstimmungsvaliditätsanalyse Korrelationsprüfungen mit nichtvalidierten Fragebögen statt; einerseits mit dem HPS [4], andererseits mit dem APHAB [49], dessen Verwendung nur für die Hörgeräte-Versorgung, nicht aber für die Cochleaimplantat-Versorgung validiert wurde. Nichtvalidierte Fragebögen sind nicht empirisch überprüft und erfüllen folglich nicht die sog. Testgütekriterien [38]. In vorherigen Studien wurde allerdings gezeigt, dass beide Fragebögen ein potenziell wertvolles klinisches Instrument sind, um die mit einem Hörverlust einhergehende Behinderung und die mit einem Cochleaimplantat erzielte Verringerung der Behinderung zu quantifizieren [23, 35, 45, 49, 51].

Die vorliegende Studie zeigt, dass die deutsche Version des NCIQ trotz der teils heterogenen Validierungsergebnisse die Berechtigung hat, in Studien zur gesundheitsbezogenen Lebensqualität bei Ertaubten im durchschnittlichen Alter von 55 Jahren vor und nach Cochleaimplantation eingesetzt zu werden. Im Rahmen der immer mehr an Bedeutung gewinnenden Sicherung der Ergebnisqualität wird die Lebensqualitätsforschung in den kommenden Jahren weiterhin zunehmendes Gewicht erhalten [2]. Die Anwendung validierter Messinstrumente ist hierfür eine unabdingbare Voraussetzung.

## Fazit für die Praxis

- Die deutsche Übersetzung des NCIQ scheint ein geeignetes Patient-Reported-Outcome-Tool zur Messung der gesundheitsbezogenen Lebensqualität vor und nach einer Cochleaimplantation zu sein.
- Validierte Lebensqualitätsmessinstrumente sind zur Sicherung der Ergebnisqualität nach medizinischen Interventionen unabdingbar.
